# Clinical implication and immunological landscape analyses of ANLN in pan‐cancer: A new target for cancer research

**DOI:** 10.1002/cam4.5177

**Published:** 2022-08-28

**Authors:** Lan Zhang, Yong Wei, Yan He, Xiaping Wang, Zebo Huang, Libing Sun, Jie Chen, Qingyi Zhu, Xin Zhou

**Affiliations:** ^1^ Department of Radiation Oncology Shanghai Tenth People's Hospital of Tongji University Shanghai China; ^2^ Department of Urology The Second Affiliated Hospital of Nanjing Medical University Nanjing China; ^3^ Department of Radiotherapy & Oncology The Affiliated Suzhou Hospital of Nanjing Medical University Suzhou China; ^4^ Department of Pathology The Second Affiliated Hospital of Nanjing Medical University Nanjing China; ^5^ Department of Oncology The Affiliated Hospital of Jiangnan University Wuxi China; ^6^ Department of Pathology The Affiliated Suzhou Hospital of Nanjing Medical University Suzhou China; ^7^ Department of Radiation Oncology Dushu Lake Hospital Affiliated to Soochow University (Medical Center of Soochow University) Suzhou China; ^8^ State Key Laboratory of Radiation Medicine and Protection Soochow University Suzhou China; ^9^ Department of Oncology First Affiliated Hospital of Nanjing Medical University Nanjing China

**Keywords:** ANLN, immune infiltration, pan‐cancer, prognosis, TMB

## Abstract

**Background:**

Anillin is a F‐actin binding protein (ANLN) mainly involved in the process of cytokinesis and known to be dysregulated in diverse cancers. However, the role of ANLN in pan‐cancer prognosis and tumor immunity remains unclear.

**Methods:**

Gene expression profiles of 31 solid tumors were downloaded from The Cancer Genome Atlas (TCGA) database. ANLN mRNA and protein expression were quantified using quantitative real‐time PCR (qRT‐PCR) and immunohistochemistry (IHC). Protein expression of ANLN was further confirmed in Human Protein Atlas (HPA) database. Cox regression and Kaplan–Meier analysis were utilized to assess the prognostic value of ANLN in pan‐cancer. The correlation between ANLN and different immune gene markers and infiltration cells was analyzed via ESTIMATE and CIBERSORT. A BLCA immunotherapy cohort: IMvigor (210) was used to confirm the role of ANLN in immune response.

**Results:**

ANLN upregulation was detected in 21 types of cancers and was associated with poor overall survival (OS), disease‐free interval (DFI), and progression‐free interval (PFI) in most cancers except in THYM (Thymoma). Additionally, correlation analysis revealed a significantly positive association between ANLN expression and tumor mutation burden (TMB), microsatellite instability (MSI), immune cells infiltration. and immune checkpoint genes in various cancers. The BLCA immunotherapy cohort confirmed that patients with higher ANLN level had better immune responses and longer OS.

**Conclusion:**

ANLN may serve as a prognostic biomarker for pan‐cancer. ANLN upregulation is associated with higher TMB, MSI, and immune cell infiltration in multiple types of tumors, shedding new light for cancer treatment.

## Graphical abstract

Upregulation of ANLN was associated with worse clinical outcomes in most solid tumors. ANLN was positively related with TMB, MSI, immune cells infiltration and immune checkpoint genes in various cancers. Higher ANLN level predicted better immune responses and longer OS in BLCA patients receiving immunotherapy.

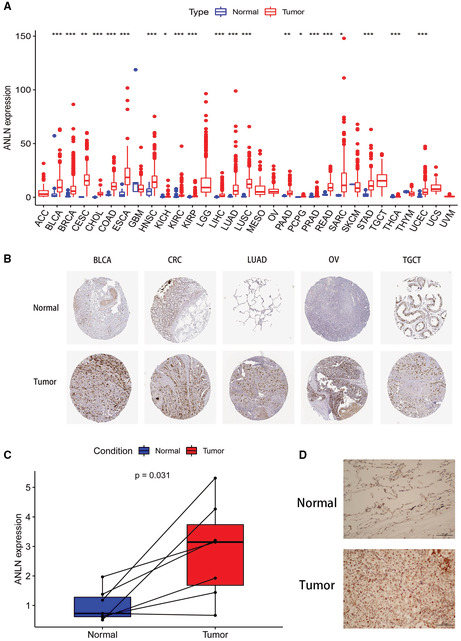

## 1 INTRODUCTION

Anillin (ANLN) is a F‐actin binding protein originally isolated from Drosophila embryo.^1^ It is highly conserved and mainly involved in the cortical cytoskeletal dynamics during cytokinesis and cellularization.^2^ Dysregulations of ANLN is observed in a wide range of malignant tumors and its overexpression is closely linked to unfavorable outcomes in colorectal cancer,^3^ breast cancer,^4^ and liver cancer.^5^ However, the role of ANLN in pan‐cancer remains still unclear.

The tumor microenvironment (TME) consists of not only cancer cells, but also tumor‐associated normal cells, including infiltrating stromal and immune cells.^6^ Increasing evidence suggests that TME has multiple effects on tumorigenesis, development, and prognosis.^7^, ^8^, ^9^, ^10^, ^11^ However, the possible mechanisms of stromal and immune components remain elusive. Immunotherapy has proved to be a powerful clinical strategy for cancer treatment.^12^ Monoclonal antibodies targeting cytotoxic T lymphocytes‐associated antigen 4 (CTLA4), programed death‐1 (PD‐1) receptor, and its ligand (PD‐L1) have achieved clinical success in some cancers.^13^, ^14^, ^15^ However, only a subset of patients could benefit from current immunotherapies.^16^ Thus, the discovery of effective immunotherapy targets is of great clinical significance.

Hence, our study comprehensively explored the prognostic significance of ANLN in 31 tumors based on The Cancer Genome Atlas (TCGA) data. Subsequently, we explored the potential correlations between ANLN and tumor immune characteristics.

## 2 METHODS

### 2.1 Data collection and preprocessing

Gene expression profiles of 31 types of solid tumors including raw counts and Fragments Per Kilobase of transcript per Million (FPKM)‐normalized RNA‐seq data were downloaded from TCGA database (https://portal.gdc.cancer.gov/) on 20 April, 2021 (ACC: adrenocortical carcinoma, BLCA: bladder urothelial carcinoma, BRCA: breast invasive carcinoma, CESC: cervical squamous cell carcinoma, CHOL: cholangiocarcinoma, COAD: colon adenocarcinoma, DLBC: lymphoid neoplasm diffuse large B‐cell lymphoma, ESCA: esophageal carcinoma, GBM: glioblastoma multiforme, LGG: brain lower grade glioma, HNSC: head and neck squamous cell carcinoma, KICH: kidney chromophobe, KIRC: kidney renal clear cell carcinoma, KIRP: kidney renal papillary cell carcinoma, LIHC: liver hepatocellular carcinoma, LUAD: lung adenocarcinoma, LUSC: lung squamous cell carcinoma, MESO: mesothelioma, OV: ovarian serous cystadenocarcinoma, PAAD: pancreatic adenocarcinoma, PCPG: pheochromocytoma and paraganglioma, PRAD: prostate adenocarcinoma, READ: rectum adenocarcinoma, SARC: sarcoma, SKCM: skin cutaneous melanoma, STAD: stomach adenocarcinoma, TGCT: testicular germ cell tumors, THCA: thyroid carcinoma, THYM: thymoma, UCEC: uterine corpus endometrial carcinoma, UCS: uterine carcinosarcoma, UVM: uveal melanoma). Acute myeloid leukemia (LAML) and large B‐cell lymphoma (DLBC) were excluded because they were hematological tumors. The primary data are presented in Table 1. Protein‐encoding genes were aligned to the human reference genome assembly GRCh38. Transcriptome files of 18,321 related genes were normalized with the assistance from R packages “limma” on R platform v3.6.3. and converged into a matrix for further analysis. The difference in ANLN expression was compared between normal tissues and tumor tissues using the Wilcoxon test with a threshold of *p* < 0.05. ANLN protein expression data in 16 types of cancers were retrieved via the Human Protein Atlas (HPA) database (https://www.proteinatlas.org/).

**TABLE 1:** Pan‐cancer data acquired from TCGA

Cancer type	Full name	Tumor samples	Normal samples
ACC	Adrenocortical carcinoma	79	0
BLCA	Bladder urothelial carcinoma	414	19
BRCA	Breast invasive carcinoma	1109	120
CESC	Cervical squamous cell carcinoma and endocervical adenocarcinoma	306	3
CHOL	Cholangiocarcinoma	36	9
COAD	Colon adenocarcinoma	480	41
ESCA	Esophageal carcinoma	162	11
GBM	Glioblastoma multiforme	169	5
HNSC	Head and neck squamous cell carcinoma	502	44
KICH	Kidney chromophobe	65	24
KIRC	Kidney renal clear cell carcinoma	539	72
KIRP	Kidney renal papillary cell carcinoma	289	32
LGG	Brain lower grade glioma	529	0
LIHC	Liver hepatocellular carcinoma	374	50
LUAD	Lung adenocarcinoma	535	59
LUSC	Lung squamous cell carcinoma	502	49
MESO	Mesothelioma	86	0
OV	Ovarian serous cystadenocarcinoma	379	0
PAAD	Pancreatic adenocarcinoma	178	4
PCPG	Pheochromocytoma and paraganglioma	183	3
PRAD	Prostate adenocarcinoma	499	52
READ	Rectum adenocarcinoma	167	10
SARC	Sarcoma	263	2
SKCM	Skin cutaneous melanoma	471	1
STAD	Stomach adenocarcinoma	375	32
TGCT	Testicular germ cell tumors	156	0
THCA	Thyroid carcinoma	510	58
THYM	Thymoma	119	2
UCEC	Uterine corpus endometrial carcinoma	552	35
UCS	Uterine carcinosarcoma	56	0
UVM	Uveal melanoma	80	0

### 2.2 Prognosis analysis

The prognostic values of ANLN, including Overall Survival (OS) and Disease‐Free Interval (DFI) and Progression‐Free Interval (PFI), was assessed via univariate Cox regression model. Samples were clustered into high or low expression cohorts based on the median value of ANLN. Kaplan–Meier (K‐M) curves and the forest plots were utilized to compare the survival time differences. All analyses were carried out using the “survival” and “survminer” package. After that, the associations between ANLN and clinical parameters, including age, sex, and the TNM stage, were assessed by the chi‐square test or Fisher's exact test. All tests were two‐tailed and a *p*‐value of 0.05 or less was considered statistically significant.

### 2.3 Correlation between ANLN expression and TMB and MSI

Tumor mutational burden (TMB) is a measure of the amount of somatic coding mutations per DNA megabase.^17^ Microsatellite instability (MSI) is a molecular tumor phenotype of a deficient mismatch repair system.^18^ Analysis regarding the correlation between ANLN expression and TMB/MSI was conducted by Pearson correlation analysis and shown in the radar chart using the “fmsb” package.

### 2.4 Correlation between ANLN expression and tumor immunity

ESTIMATE (Estimation of Stromal and Immune cells in Malignant Tumor tissues using Expression data) is a method for estimating the presence of stromal/immune cells in tumor tissues and tumor purity with high accuracy.^19^ This method was applied to explore the association between the immune and stromal cell ratio with ANLN expression.^19^

We further explored the correlation between ANLN expression and 22 types of infiltrating immune cells using the “CIBERSORT” algorithm. The association between ANLN expression and common immune checkpoint genes was investigated using the “Reshape2” package and shown in heatmaps by the R “RColorBrewer” package. The Spearman method was utlized to assess the correlation coefficients.

### 2.5 Collection of an immunotherapy‐based cohort

After systematically searching for immunotherapy gene expression profiles, an immunotherapeutic cohort: advanced urothelial cancer with intervention of atezolizumab, an anti‐PD‐L1 antibody (IMvigor210 cohort) with complete clinical information was recruited in our study.^20^ The complete information of the cohort is available on http://research‐pub.Gene.com/imvigor210corebiologies. The raw count data were normalized by the DEseq2 R package and transformed into the TPM value for further study.

### 2.6 Gene set enrichment analysis

Next, our study explored the potential molecular functions and biological pathways regulated by ANLN. The Gene Set Enrichment Analysis (GSEA) software (http://www.broadinstitute.org/gsea) was used to investigate the enrichment of GO and KEGG pathways between high ANLN expression and low expression groups according to the median value of ANLN. All analyses were conducted with R package “org.Hs.eg.db,” “clusterProfiler,” “enrichplot,” “DOSE/limma.” Gene sets with |NES| > 1, NOM *p* < 0.05, and FDR q < 0.25 were considered as enrichment significant.

### 2.7 Experimental validation

Fresh tissue samples of 7 LUAD patients were obtained from the Affiliated Suzhou Hospital of Nanjing Medical University. The mRNA and protein expression of ANLN were quantified using quantitative real‐time PCR (qRT‐PCR) and immunohistochemistry (IHC) as previously described.^10^ Briefly, qRT‐PCR was conducted on a LightCycler 480 (Roche 480, Germany) real‐time thermal cycler, using SYBR Green dye. Each sample was set with three multiple wells. Assays detected with 5 Ct's less than the negative control (No Template Control, NTC), and with Ct < 35 were included for further data analysis. The melting analysis was added finally to evaluate the specificity of PCR products. Expression levels of ANLN were calculated using 2^−ΔΔCt^ method and normalized to that of GAPDH mRNA (forward: 5′‐GTCTCCTCTGACTTCAACAGCG −3′ and reverse: 5′‐ACCACCCTGTTGCTGTAGCCAA −3′). The sequences of the primers used were as follows: forward: 5′‐CAGACAGTTCCATCCAAGGGAG‐3′ and reverse: 5′‐CTTGACAACGCTCTCCAAAGCG‐3′. IHC staining for ANLN was accomplished by two professional pathologists. Formalin‐fixed human lung tumor tissue sections were sliced into serial sections with a thickness of 5 μm for IHC. An anti‐ANLN antibody (anti‐ANLN: catalog no., BS‐7738R; dilution, 1:200; Bioss Inc.) was used as the primary antibody. PBS was functioned a negative control to keep the specificity of staining. A 3,3′‐diaminobenzidine (DAB) and hematoxylin counterstain (ZSGB‐Bio, China) were used to visualize antibody staining. Finally, immunostained sections were photographed using a microscope (Olympus Corporation). This study was approved by the Ethics Committee of the hospital. Each participant had signed informed consent in accordance with the Declaration of Helsinki (Ethics Approval No. KL901198).

## 3 RESULTS

### 3.1 Pan‐cancer ANLN expression

**FIGURE 1:**
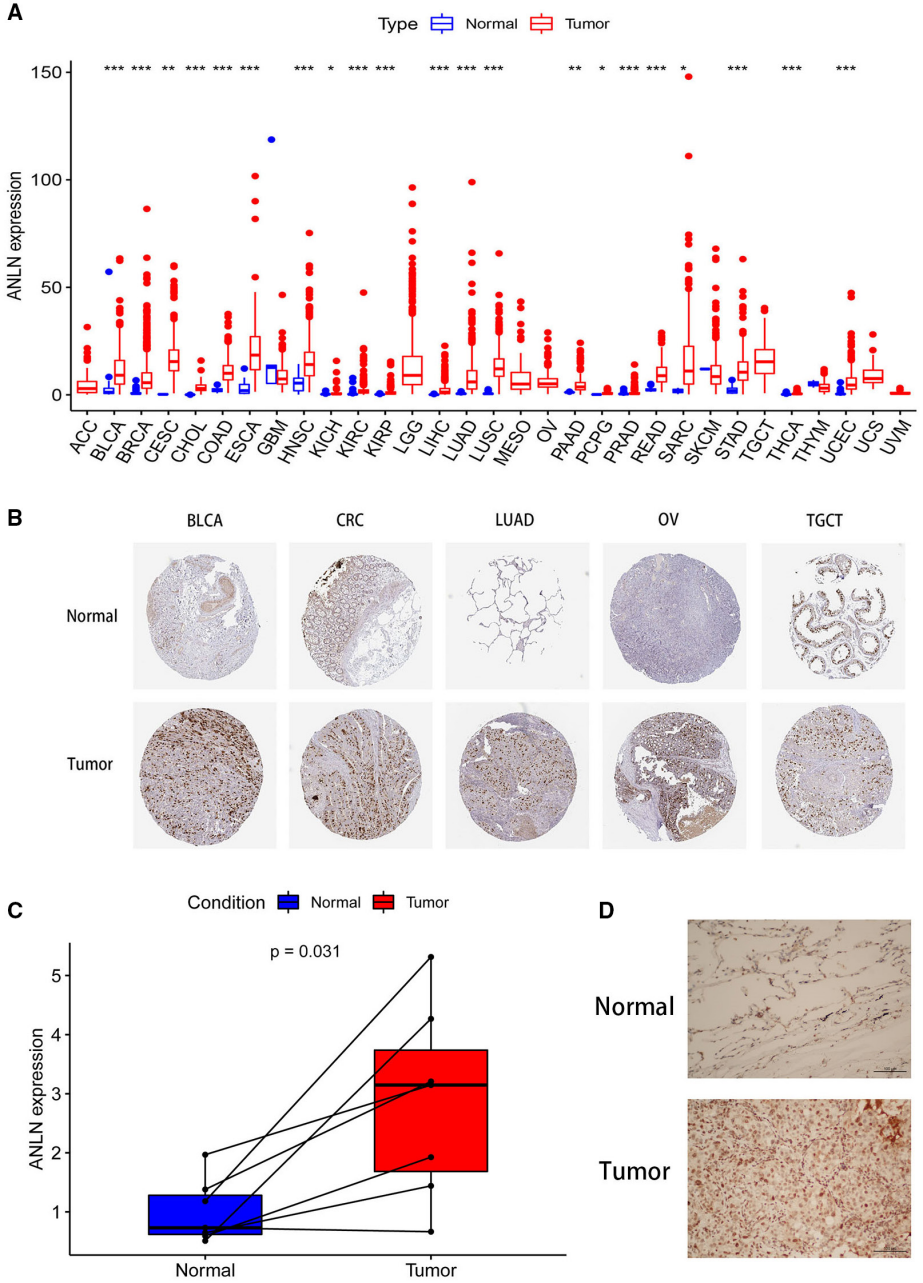
ANLN expression in pan‐cancer. (A) Boxplot of the gene expression of ANLN in cancers and normal tissues from The Cancer Genome Atlas (TCGA) database. (B) Representative immunohistochemical staining of ANLN in colon, LUAD, PRAD normal and cancer samples, data from The Human Protein Atlas (THPA) database. *p < 0.05; **p < 0.01, ***p < 0.001, ****p < 0.0001. (C) MRNA Expression level of ANLN was higher in LUAD tissues than those in normal tissues. (D) IHC analysis showed that protein level of ANLN was higher in LUAD tissues. Representative images are shown.

Our study firstly analyzed the expression pattern of ANLN in tumor and normal samples from the TCGA pan‐cancer dataset. As shown in Figure 1A, ANLN expression was significantly increased in BLCA, BRCA, CESC, CHOL, COAD, ESCA, HNSC, KICH, KIRC, KIRP, LIHC, LUAD, LUSC, PAAD, PCPG, PRAD, READ, SARC, STAD, THCA, and UCEC. Next, we investigated ANLN protein expression based on the HPA database. ANLN expression was significantly higher in tumor samples than normal tissues in BLCA, CRC (including COAD and READ), LUAD, OV, and TGCT (Figure 1B). We further validated ANLN expression in LUAD patients. The immunohistochemical and mRNA results demonstrated that ANLN expression was significantly higher in tumor tissues than paracancerous samples (Figure 1C,D).

### 3.2 Survival analysis of ANLN

**FIGURE 2:**
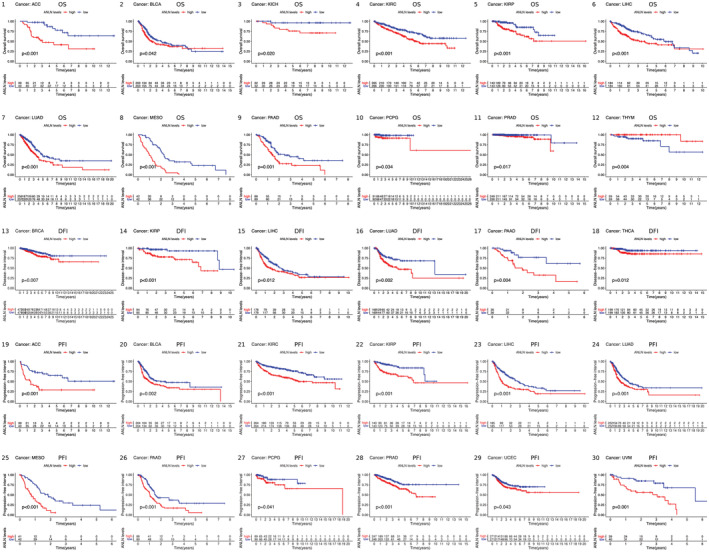
Kaplan–Meier survival curves comparing ANLN expression in pan‐cancer. (1–12): Kaplan–Meier curves for patients' OS (overall survival) in 12 tumor types; (13–18): Kaplan–Meier curves for patients' DFI (disease‐free interval) in 6 tumor types; (19–30): Kaplan–Meier curves for patients' PFI (progression‐free interval) in 12 tumor types. *p* < 0.05 was considered to be significant.

Our study investigated the correlation between ANLN expression and patients' OS, DFI, PFI using the TCGA cohort. Kaplan–Meier curves showed that high expression of ANLN was significantly associated with poor OS in ACC, BLCA, KICH, KIRC, KIRP, LIHC, LUAD, MESO, PAAD, PCPG, and PRAD. Particularly, low expression of ANLN was linked to poor OS in THYM. In addition, high ANLN expression was correlated with poor DFI in BRCA, KIRP, LIHC, LUAD, PAAD, and THCA. Results of the Kaplan–Meier plot also suggested that increased level of ANLN was distinctly connected with worse PFI in patients with ACC, BLCA, KIRC, KIRP, LIHC, LUAD, MESO, PAAD, PCPG, PRAD, UCEC, and UVM (Figure 2).

**FIGURE 3:**
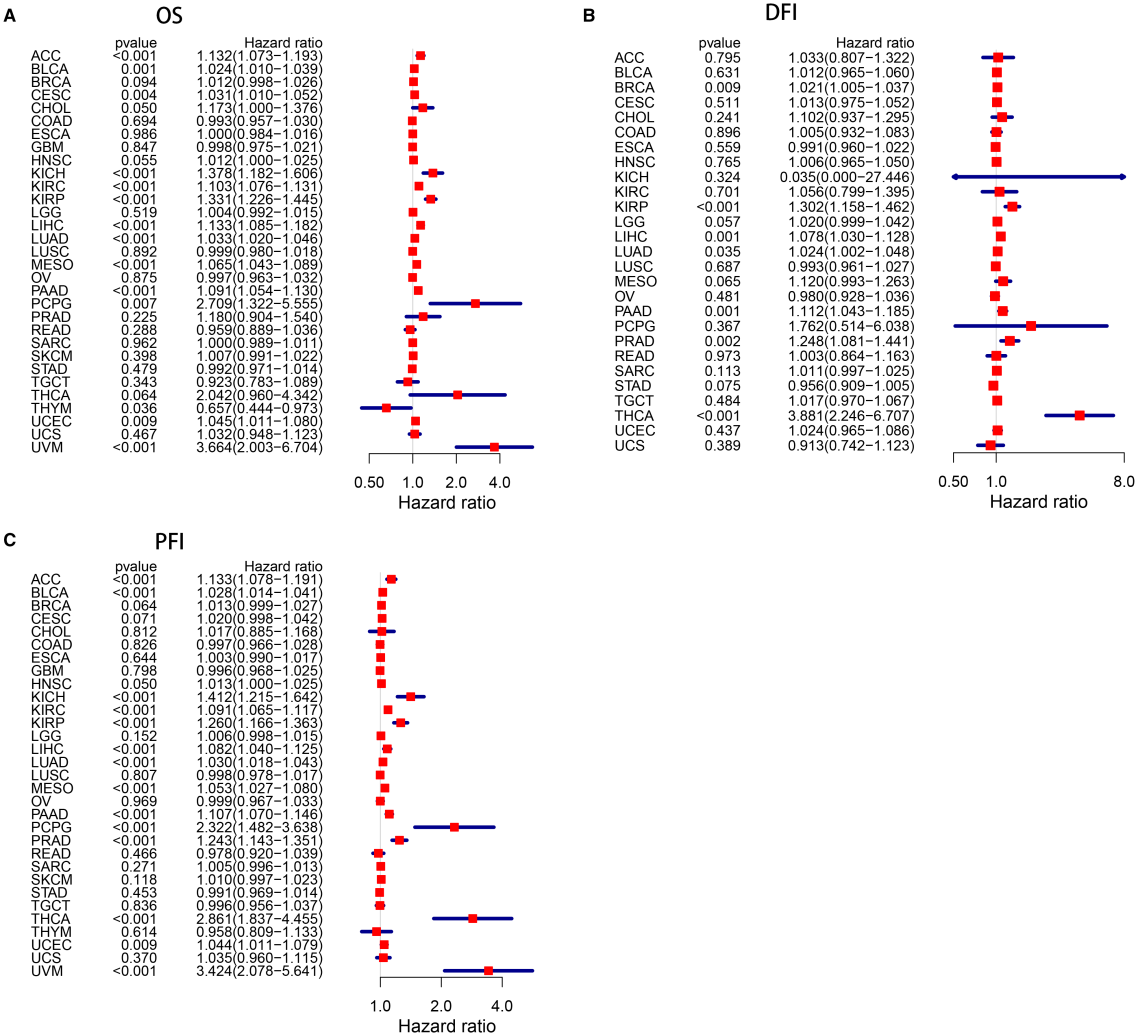
Forest plots of hazard ratios of ANLN in pan‐cancer. (A) Association between ANLN expression and OS; (B) Association between ANLN expression and DFI; (C) Association between ANLN expression and PFI. Cox regression analysis. *p* < 0.05 was considered to be significant.

In Cox regression analysis, high ANLN expression was an unfavorable factor for patients' OS in ACC, BLCA, CESC, CHOL, KICH, KIRC, KIRP, LIHC, LUAD, MESO, PAAD, PCPG, UCEC, and UVM. While patients with low ANLN expression showed shortened OS in THYM. For DFI, elevated level of ANLN was significantly correlated with shorter DFI in BRCA, KIRP, LIHC, LUAD, PAAD, PRAD, and THCA. Additionally, increased level of ANLN was strongly correlated with worse PFI in ACC, BLCA, HNSC, KICH, KIRC, KIRP, LIHC, LUAD, MESO, PAAD, PCPG, PRAD, THCA, UCEC, and UVM (Figure 3).

**FIGURE 4:**
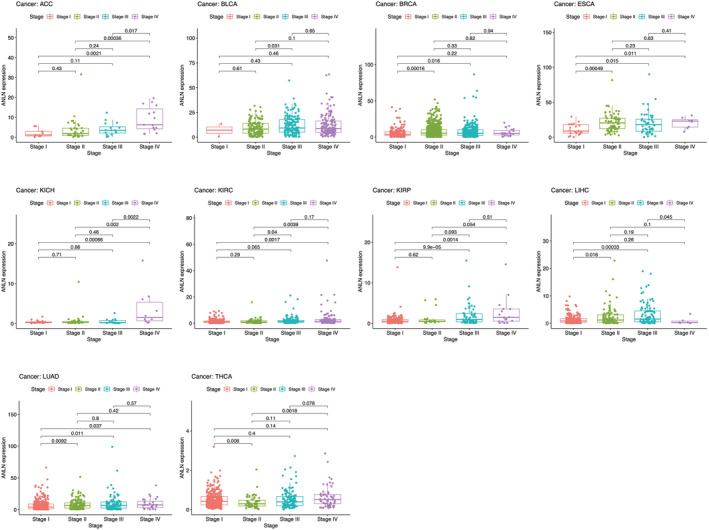
Association between ANLN expression and different TNM stages in patients with ACC, BLCA, BRCA, ESCA, KICH, KIRC, KIRP, LUAD, LIHC, and THCA. *p* < 0.05 was considered to be significant.

Furthermore, we explored the correlation between ANLN expression and different clinical features across pan‐cancer. Results revealed that higher expression of ANLN was significantly associated with advanced TNM stage in ACC, BLCA, BRCA, ESCA, KICH, KIRC, KIRP, LUAD, and reversely in LIHC. Interestingly, ANLN expression was higher in stage I compared with stage II (*p* = 0.008), but lower in stage II compared with stage IV (*p* = 0.0018) in THCA (Figure 4). ANLN expression also differs greatly in age and gender across different tumors (Figure S1).

### 3.3 Tumor mutational burden and microsatellite instability analysis data

**FIGURE 5:**
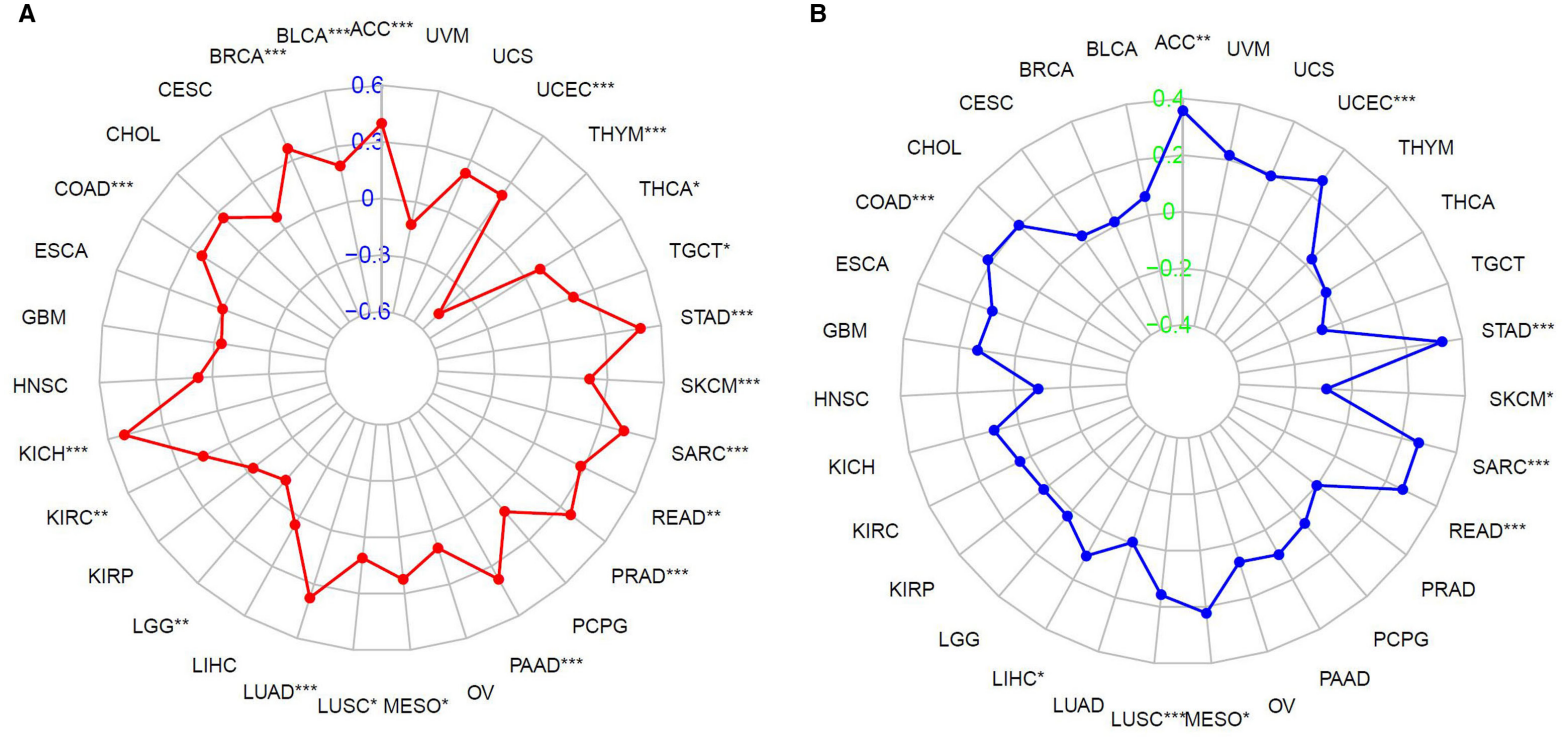
Associations between ANLN expression and tumor mutation burden (TMB) and microsatellite instability (MSI) across pan‐cancer. (A) Association between ANLN expression and TMB; (B) Association between ANLN expression and MSI. *p < 0.05, **p < 0.01, ***p < 0.001.

TMB and MSI are essential in the process of tumor formation and development. We assessed the association between ANLN expression and TMB/MSI across 31 cancers. Results suggested that elevated ANLN expression was significantly associated with higher TMB in ACC, BLCA, BRCA, COAD, KICH, KIRC, LUAD, LUSC, MESO, PAAD, PRAD, READ, SARC, SKCM, STAD, TGCT, THCA, and UCEC, while increased ANLN level was inversely related to TMB in LGG and THYM (Figure 5). In addition, ANLN expression was positively associated with MSI in ACC, COAD, LIHC, LUSC, MESO, READ, SARC, STAD, UCEC, and negatively linked to SKCM (Figure 5).

### 3.4 Correlation between ANLN expression and immune infiltration

**FIGURE 6:**
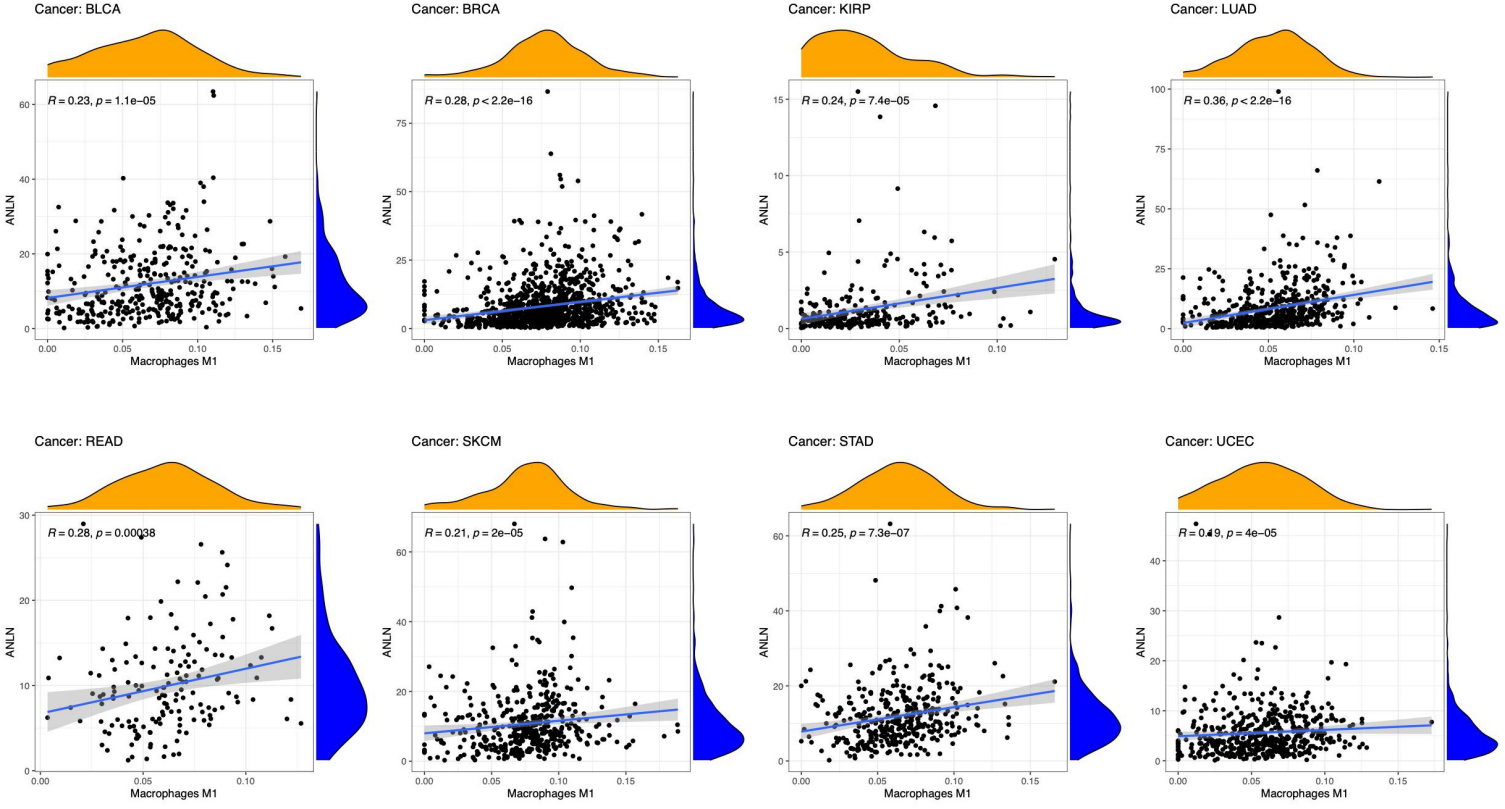
Association between ANLN expression and Macrophages M1 in patients with BLCA, BRCA, KIRP, LUAD, READ, SKCM, STAD, and UCEC (*p* < 0.00001).

Tumor‐infiltrating immune cells are important components for the tumor microenvironment, and are closely linked to tumorigenesis and progression.^21^ Infiltrating stromal and immune cells in tumor microenvironment play essential roles in disturbing tumor signaling and cancer biology.^6^ Hence, we firstly explored the correlation between ANLN expression and tumor‐infiltrating immune cells. Our results demonstrated that ANLN expression was significantly associated with the infiltration levels of macrophages M1 in 18 cancer types (Figure 6, those with *p* < 0.00001 were shown), CD4 + T cells in 12 cancer types, CD8+ T cells in 9 cancer types, T cells regulatory (Tregs) in 13 cancer types, B cells in 8 cancer types, M2 in 7 cancer types, NK cells in 9 cancer types, and neutrophils in 7 cancer types (those with *p* < 0.00001 were shown in Figure S2). For instance, in BLCA, ANLN expression was positively correlated with M1 macrophages and neutrophil infiltration, but negatively correlated with Tregs. In HNSC, ANLN expression was negatively correlated with CD8+ cells, Tregs, but positively correlated with CD4 + T cells memory resting.

**FIGURE 7:**
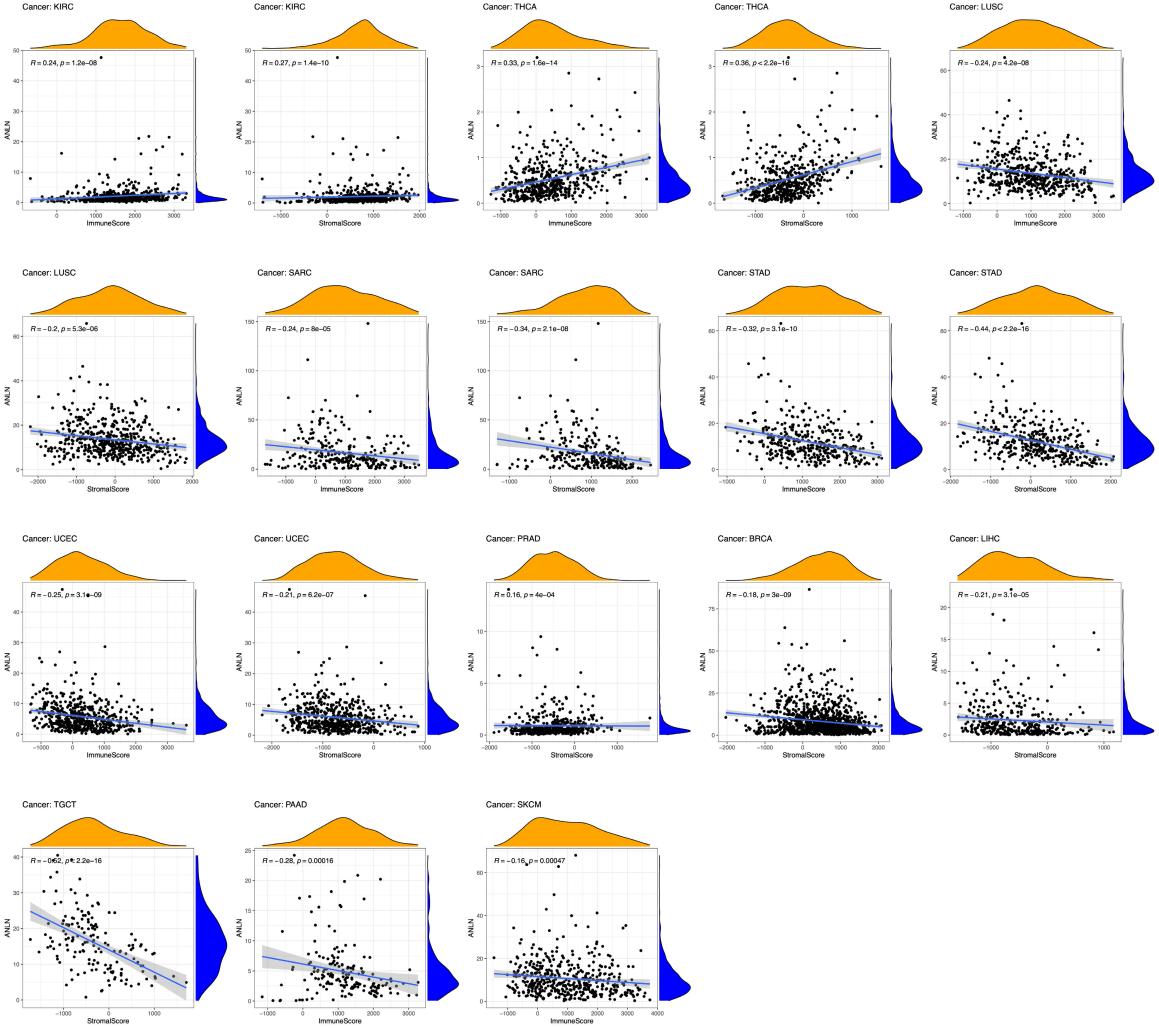
Association between ANLN expression and the immune and stromal cell ratio in patients' with KIRC, THCA, LUSC, SARC, STAD, UCEC, PRAD, BRCA, LIHC, TGCT, PAAD, and SKCM. *p* < 0.05 was considered to be significant.

Secondly, our study investigated the association between ANLN expression and the immune and stromal cell ratio. Results showed that ANLN expression is positively correlated with the stromal score and immune score in KIRC, THCA, while negatively correlated with in LUSC, SARC, STAD, and UCEC (Figure 7). In addition, our study observed a statistical positive connection between ANLN expression and stromal score in PRAD but noted a negative connection in BRCA, LIHC, TGCT. Moreover, ANLN expression was negatively linked to immune score in PAAD and SKCM (Figure 7).

**FIGURE 8:**
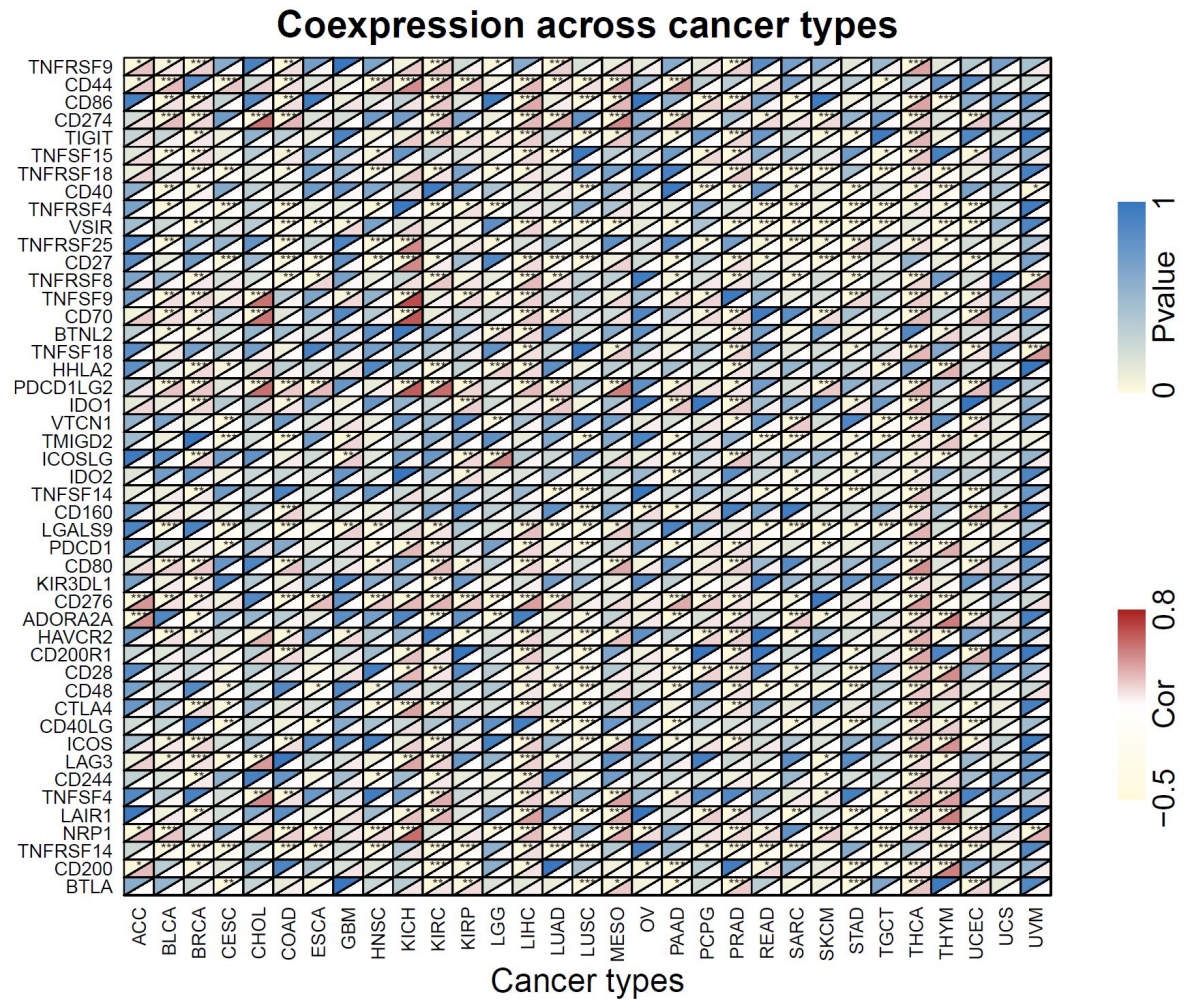
Association between ANLN expression and 47 common immune checkpoints gene levels in pan‐cancer. *p < 0.05, **p < 0.01, ***p < 0.001.

Immune checkpoint genes (ICGs) represent a novel target in cancer treatment. Therefore, we further examined the association between ANLN expression and the common immune checkpoint genes. As shown in Figure 8, cytotoxic T lymphocyte‐associated antigen 4 (CTLA‐4), programed death 1 (PD‐1), and PD‐L1 were positively associated with ANLN expression in multiple cancers, including BRCA, KICH, KIRC, LIHC, PRAD, and THCA. On the contrary, there was a negative connection between ANLN expression and CTLA‐4, PD‐1 in CESC, HNSC, and LUSC. Moreover, ANLN expression was positively linked to the majority of immune checkpoint genes in THCA. However, ANLN expression demonstrated a negative correlation with most of the immune checkpoint genes in GBM, UCS, and UVM.

### 3.5 Immunotherapeutic response prediction

**FIGURE 9:**
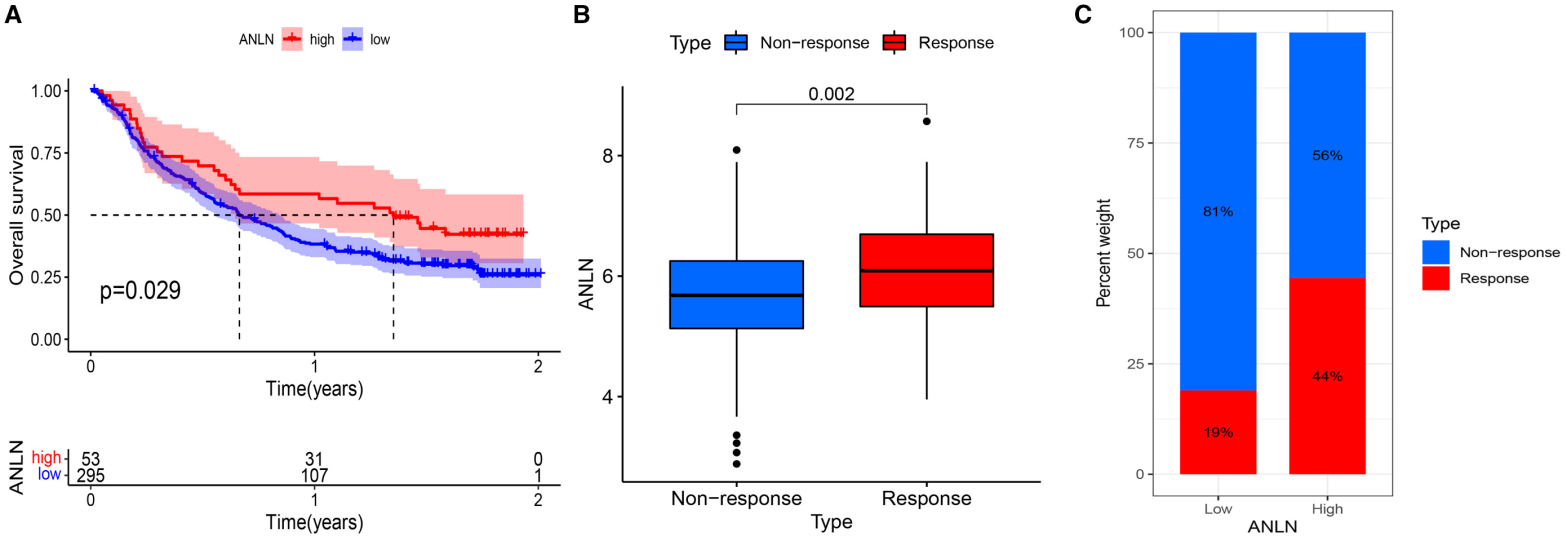
ANLN expression in the role of immunotherapeutic response prediction. (A) Kaplan–Meier curves for patients' OS; (B) Differences in ANLN expression between non‐response and response types; (C) The proportion of patients with response to PD‐L1 blockade immunotherapy in low or high ANLN groups. *p* < 0.05 was considered to be significant.

Considering the strong correlation between ANLN expression and tumor immune landscape, our study next explored the immunotherapeutic predictive ability of ANLN in an anti‐PD‐L1 cohort (IMvigor210). The Kaplan–Meier curves suggested that increased level of ANLN was significantly connected with prolonged OS (Figure 9A). In addition, patients with higher ANLN expression level exhibited better immune response (Figure 9B,C).

### 3.6 GO and KEGG‐based gene set enrichment analysis

Next, GSEA was applied to explore the functional enrichment of ANLN expression between high expression and low expression cohorts. GO enrichment term demonstrated that high expression of ANLN was mainly associated with cell immunity and cell proliferation (Figure S3). KEGG enrichment term exhibited that high expression of ANLN was positively involved “cell cycle” pathways in ACC, BRCA, KICH, HHSC, KICH, LUAD. MESO, OV, PAAD, PCPG, SARC, and UVM (Figure S4).

## 4 DISCUSSION

The pan‐cancer analysis project launched by TCGA was aimed to illustrate an integrated picture of commonalities and heterogeneities across different tumors.^22^ The project interpreted molecular aberrations from genomic, transcriptional, proteomic, and epigenetic levels in large patient cohorts, providing comprehensive information for early diagnosis and treatment strategy.^23^ Our study is the first to comprehensively analyze the expression pattern of ANLN and its correlation with patients' prognosis and immune features in 31 different types of cancers.

Our study found that ANLN was upregulated in BLCA, BRCA, CESC, CHOL, COAD, ESCA, HNSC, KICH, KIRC, KIRP, LIHC, LUAD, LUSC, PAAD, PCPG, PRAD, READ, SARC, STAD, THCA, and UCEC. Cox regression and Kaplan–Meier analysis demonstrated that high ANLN expression was associated with shorter OS, DFI, and PFS in KIRP, LIHC, LUAD, and PAAD. This is in line with a recent study that ANLN is a prognostic biomarker for LUAD patients.^24^ ANLN is primarily involved in the process of cytokinesis and plays vital roles in tumorigenesis and progression.^2^ ANLN could significantly promote cancer cell growth and migration via targeting miR‐217^25^ and HMGA2.^26^ Upregulation of ANLN could activate RHOA, leading to the activation of phosphatidylinositol 3‐kinase‐AKT (PI3K/AKT), which promotes cell proliferation and progression.^27^ Another study revealed that ANLN might be involved in the metastasis of lung adenocarcinoma by promoting epithelial mesenchymal transformation of tumor cells.^28^ In addition, ANLN expression is also positively related to Wnt/β‐catenin signaling in gastric cancer.^29^ All these biological functions of ANLN may explain its oncogenic role in the above‐mentioned cancers. Notably, over‐expression of ANLN was correlated with better prognosis in THYM. Future studies are needed to explore its controversial role in human cancer.

TMB is emerging as a promising biomarker for immunotherapy. It refers to the total number of somatic coding mutations, gene insertion, base substitutions, and deletion errors detected per million bases.^30^ TMB presents as a useful estimation of tumor neoantigens, which could activate tumor‐specific T cells to recognize and kill tumor cells.^30^ High TMB are associated with prolonged survival in melanoma,^31^ NSCLC,^32^ and breast cancer.^33^ MSI is a molecular tumor phenotype caused by genomic hypermutability.^34^ Recent studies suggested that MSI could serve as an efficient biomarker for immunotherapy.^35^ Clinical trials showed that MSI‐H patients have better outcomes than microsatellite stable patients, owning to the activation of T lymphocyte recognition of neoantigens.^35^, ^36^ Additionally, nearly all patients with MSI‐H have high TMB level and could respond more strongly to immunotherapy.^17^ Our study found that ANLN expression was positively associated with TMB or MSI in multiple cancers, including BLCA, which was consistent with the results from the BLCA immunotherapy cohort that patients with higher ANLN level had better immune responses and thus demonstrated longer OS. All the above findings suggested that ANLN expression in these tumors may help identify patients who are more likely to benefit from immunotherapy.

TME is a complex ecology consisting of tumor cells, tumor vasculature, surrounding immune and inflammatory cells, as well as adipocytes and fibroblasts.^6^ Tumor immune microenvironment (iTME), as a core part of the TME, consists of tumor‐infiltrating lymphocytes (TILs) and other immune cells, such as macrophages, neutrophils, and dendritic cells.^37^ Macrophages is a group of heterogeneous cells in the iTME and is strongly correlated with poor survival in diverse cancers.^38^ Tumor‐associated macrophages (TAMs) play essential roles in carcinogenesis and progression by providing cytokines and inducing tumor angiogenesis.^39^ TAMs could be polarized into two distinct phenotypes: (1) M1 macrophages, which act as an anti‐tumor defender; (2) M2 macrophages, which could promote tumor invasion and metastasis.^39^ Our study found that ANLN expression was positively associated with M1 macrophage infiltration, but negatively linked to M2 macrophage infiltration. Thus, we assumed that ANLN might participate in the host anti‐tumor immune response by inducing M1 macrophages infiltration. However, we also found that ANLN expression was negatively connected to CD8+ T cells in some cancer types. Cytotoxic CD8+ memory T cells in the TME is positively linked to cancer prognosis in diverse cancers.^40^, ^41^, ^42^ It could contribute to cancer prognoses by killing tumor cells.^43^ This phenomenon could be explained by the existence of other important regulators in the interaction between ANLN and CD8+ T cells. Further studies are needed to investigate the exact mechanism during this anti‐tumor progress. Single‐cell RNA sequencing (scRNA‐seq), which allows for transcriptome‐wide analyses of individual cells may help offer new insights in the future. A previous study showed that ANLN along with other regulators are significantly associated with the expression of naïve B cells, regulatory T cells, and neutrophils in head and neck squamous cell carcinoma (HNSCC).^44^ However, studies focusing on the functional mechanism of ANLN in immune cell infiltration is rare. Our GO and KEGG pathway analyses revealed that ANLN was mainly enriched in cell cycle, immunity, and metabolic‐related pathway, which may explain its oncogenic role in different tumors. Further studies are needed to find the exact functional mechanism between ANLN expression and different immune cells. Moreover, our analyses demonstrated that ANLN expression had a significant correlation with most of the common immune checkpoint markers. We further validated the immunotherapeutic predictive ability of ANLN in an anti‐PD‐L1 cohort (IMvigor210). Results showed that high ANLN expression was associated with prolonged OS and better immune response, which was consistent with the above definitions. All these above findings confirmed our hypothesis that ANLN could be a robust biomarker for prognosis and immune response prediction.

Several limitations should be considered in our study. First, because of the use of microarray and sequencing data from different databases, systematic bias may occur. Hence, as mentioned above, scRNA‐seq or other higher resolution methods could be performed. Second, our study was a retrospective study, so future prospective researches are needed to verify the correlation between ANLN expression and patients' prognosis and tumor immunity. Finally, the precise mechanisms by which ANLN facilitates tumor progression and tumor immunity remain largely obscure. Further mechanistic studies on ANLN at both cellular and molecular levels are needed to fully elucidate its functions.

In conclusion, our study found that ANLN was upregulated in diverse cancers and its aberrant expression was associated with pan‐cancer prognosis, MSI, TMB, the tumor immune microenvironment, and immune checkpoint genes. Therefore, ANLN may prove to be a promising biomarker for prognostic judgment and immunotherapy.

## AUTHOR CONTRIBUTIONS

JC, QYZ, and XZ conceived and designed this study. LZ, YW, and YH obtained the data. XZ, LZ, YW, and YH analyzed the data. XPW and ZBH helped discuss the results. LZ drafted the manuscript. All authors contributed to the article and approved the submitted version.

## CONFLICT OF INTEREST

The authors declare that there are no conflicts of interest.

## ETHICS APPROVAL AND CONSENTS TO PARTICIPATE

This study was approved by the Ethics Committee of The Affiliated Suzhou Hospital of Nanjing Medical University and each participant had signed informed consent (Ethics Approval No. KL901198).

## Supporting information


Figure S1

Figure S2

Figure S3

Figure 4

## Data Availability

The data that support the findings of this study are available from the corresponding author upon reasonable request.

## References

[1] Tuan NM , Lee CH . Role of anillin in tumour: from a prognostic biomarker to a novel target. Cancers (Basel). 2020;12(6):1600. 32560530 10.3390/cancers12061600 PMC7353083

[2] Chen A , Akhshi TK , Lavoie BD , Wilde A . Importin beta2 mediates the Spatio‐temporal regulation of anillin through a noncanonical nuclear localization signal. J Biol Chem. 2015;290:13500‐13509. 25829492 10.1074/jbc.M115.649160 PMC4505596

[3] Wang G , Shen W , Cui L , Chen W , Hu X , Fu J . Overexpression of anillin (ANLN) is correlated with colorectal cancer progression and poor prognosis. Cancer Biomark. 2016;16:459‐465. 27062703 10.3233/CBM-160585 PMC13016496

[4] Dai X , Mei Y , Chen X , Cai D . ANLN and KDR are jointly prognostic of breast cancer survival and can be modulated for triple negative breast cancer control. Front Genet. 2019;10:790. 31636652 10.3389/fgene.2019.00790 PMC6788326

[5] Zhou Z , Li Y , Hao H , et al. Screening hub genes as prognostic biomarkers of hepatocellular carcinoma by bioinformatics analysis. Cell Transplant. 2019;28:76S‐86S. 31822116 10.1177/0963689719893950 PMC7016461

[6] Fridman WH , Pages F , Sautes‐Fridman C , Galon J . The immune contexture in human tumours: impact on clinical outcome. Nat Rev Cancer. 2012;12:298‐306. 22419253 10.1038/nrc3245

[7] Ge Q , Li G , Chen J , et al. Immunological role and prognostic value of APBB1IP in Pan‐cancer analysis. J Cancer. 2021;12:595‐610. 33391455 10.7150/jca.50785 PMC7738982

[8] Liu J , Zhang S , Dai W , Xie C , Li JC . A comprehensive prognostic and immune analysis of SLC41A3 in Pan‐cancer. Front Oncol. 2020;10:586414. 33520701 10.3389/fonc.2020.586414 PMC7841432

[9] Xu WX , Zhang J , Hua YT , Yang SJ , Wang DD , Tang JH . An integrative Pan‐cancer analysis revealing LCN2 as an oncogenic immune protein in tumor microenvironment. Front Oncol. 2020;10:605097. 33425761 10.3389/fonc.2020.605097 PMC7786136

[10] Yang C , Wu T , Zhang J , et al. Prognostic and immunological role of mRNA ac4C regulator NAT10 in Pan‐cancer: new territory for cancer research? Front Oncol. 2021;11:630417. 34094911 10.3389/fonc.2021.630417 PMC8170476

[11] Li X , Gao Y , Xu Z , Zhang Z , Zheng Y , Qi F . Identification of prognostic genes in adrenocortical carcinoma microenvironment based on bioinformatic methods. Cancer Med. 2020;9:1161‐1172. 31856409 10.1002/cam4.2774 PMC6997077

[12] Conway JR , Kofman E , Mo SS , Elmarakeby H , Van Allen E . Genomics of response to immune checkpoint therapies for cancer: implications for precision medicine. Genome Med. 2018;10:93. 30497521 10.1186/s13073-018-0605-7 PMC6264032

[13] Gadgeel S , Rodriguez‐Abreu D , Speranza G , et al. Updated analysis from KEYNOTE‐189: pembrolizumab or placebo plus pemetrexed and platinum for previously untreated metastatic nonsquamous non‐small‐cell lung cancer. J Clin Oncol. 2020;38:1505‐1517. 32150489 10.1200/JCO.19.03136

[14] Kato K , Shah MA , Enzinger P , et al. KEYNOTE‐590: phase III study of first‐line chemotherapy with or without pembrolizumab for advanced esophageal cancer. Future Oncol. 2019;15:1057‐1066. 30735435 10.2217/fon-2018-0609

[15] Andre T , Shiu KK , Kim TW , et al. Pembrolizumab in microsatellite‐instability‐high advanced colorectal cancer. N Engl J Med. 2020;383:2207‐2218. 33264544 10.1056/NEJMoa2017699

[16] Topalian SL , Drake CG , Pardoll DM . Immune checkpoint blockade: a common denominator approach to cancer therapy. Cancer Cell. 2015;27:450‐461. 25858804 10.1016/j.ccell.2015.03.001 PMC4400238

[17] Chalmers ZR , Connelly CF , Fabrizio D , et al. Analysis of 100,000 human cancer genomes reveals the landscape of tumor mutational burden. Genome Med. 2017;9:34. 28420421 10.1186/s13073-017-0424-2 PMC5395719

[18] Thibodeau SN , Bren G , Schaid D . Microsatellite instability in cancer of the proximal colon. Science. 1993;260:816‐819. 8484122 10.1126/science.8484122

[19] Yoshihara K , Shahmoradgoli M , Martinez E , et al. Inferring tumour purity and stromal and immune cell admixture from expression data. Nat Commun. 2013;4:2612. 24113773 10.1038/ncomms3612 PMC3826632

[20] Mariathasan S , Turley SJ , Nickles D , et al. TGFbeta attenuates tumour response to PD‐L1 blockade by contributing to exclusion of T cells. Nature. 2018;554:544‐548. 29443960 10.1038/nature25501 PMC6028240

[21] Savas P , Salgado R , Denkert C , et al. Clinical relevance of host immunity in breast cancer: from TILs to the clinic. Nat Rev Clin Oncol. 2016;13:228‐241. 26667975 10.1038/nrclinonc.2015.215

[22] Cancer Genome Atlas Research N , Weinstein JN , Collisson EA , et al. The cancer genome atlas Pan‐cancer analysis project. Nat Genet. 2013;45:1113‐1120. 24071849 10.1038/ng.2764 PMC3919969

[23] Consortium ITP‐CAoWG . Pan‐cancer analysis of whole genomes. Nature. 2020;578:82‐93. 32025007 10.1038/s41586-020-1969-6 PMC7025898

[24] Song C , Wu Z , Wang Q , et al. A combined two‐mRNA signature associated with PD‐L1 and tumor mutational burden for prognosis of lung adenocarcinoma. Front Cell Dev Biol. 2021;9:634697. 33585490 10.3389/fcell.2021.634697 PMC7875126

[25] Idichi T , Seki N , Kurahara H , et al. Regulation of Actin‐binding protein ANLN by antitumor miR‐217 inhibits cancer cell aggressiveness in pancreatic ductal adenocarcinoma. Oncotarget. 2017;8:53180‐53193. 28881803 10.18632/oncotarget.18261 PMC5581102

[26] Guo HH , Wang YZ , Zhang ZK , Li MZ , Tian XD , Yang YM . High mobility group AT‐hook 2 promotes tumorigenicity of pancreatic cancer cells via upregulating ANLN. Exp Cell Res. 2020;393:112088. 32413362 10.1016/j.yexcr.2020.112088

[27] Suzuki C , Daigo Y , Ishikawa N , et al. ANLN plays a critical role in human lung carcinogenesis through the activation of RHOA and by involvement in the phosphoinositide 3‐kinase/AKT pathway. Cancer Res. 2005;65:11314‐11325. 16357138 10.1158/0008-5472.CAN-05-1507

[28] Xu J , Zheng H , Yuan S , et al. Overexpression of ANLN in lung adenocarcinoma is associated with metastasis. Thorac Cancer. 2019;10:1702‐1709. 31268619 10.1111/1759-7714.13135 PMC6669805

[29] Pandi NS , Manimuthu M , Harunipriya P , Murugesan M , Asha GV , Rajendran S . In silico analysis of expression pattern of a Wnt/beta‐catenin responsive gene ANLN in gastric cancer. Gene. 2014;545:23‐29. 24809965 10.1016/j.gene.2014.05.013

[30] Samstein RM , Lee CH , Shoushtari AN , et al. Tumor mutational load predicts survival after immunotherapy across multiple cancer types. Nat Genet. 2019;51:202‐206. 30643254 10.1038/s41588-018-0312-8 PMC6365097

[31] Kang K , Xie F , Mao J , Bai Y , Wang X . Significance of tumor mutation burden in immune infiltration and prognosis in cutaneous melanoma. Front Oncol. 2020;10:573141. 33072607 10.3389/fonc.2020.573141 PMC7531222

[32] Gandara DR , Paul SM , Kowanetz M , et al. Blood‐based tumor mutational burden as a predictor of clinical benefit in non‐small‐cell lung cancer patients treated with atezolizumab. Nat Med. 2018;24:1441‐1448. 30082870 10.1038/s41591-018-0134-3

[33] Thomas A , Routh ED , Pullikuth A , et al. Tumor mutational burden is a determinant of immune‐mediated survival in breast cancer. Onco Targets Ther. 2018;7:e1490854. 10.1080/2162402X.2018.1490854 PMC6207420 30386679

[34] Hause RJ , Pritchard CC , Shendure J , Salipante SJ . Classification and characterization of microsatellite instability across 18 cancer types. Nat Med. 2016;22:1342‐1350. 27694933 10.1038/nm.4191

[35] Le DT , Uram JN , Wang H , et al. PD‐1 blockade in tumors with mismatch‐repair deficiency. N Engl J Med. 2015;372:2509‐2520. 26028255 10.1056/NEJMoa1500596 PMC4481136

[36] Timmermann B , Kerick M , Roehr C , et al. Somatic mutation profiles of MSI and MSS colorectal cancer identified by whole exome next generation sequencing and bioinformatics analysis. PLoS One. 2010;5:e15661. 21203531 10.1371/journal.pone.0015661 PMC3008745

[37] Hinshaw DC , Shevde LA . The tumor microenvironment innately modulates cancer progression. Cancer Res. 2019;79:4557‐4566. 31350295 10.1158/0008-5472.CAN-18-3962 PMC6744958

[38] Nielsen SR , Schmid MC . Macrophages as key drivers of cancer progression and metastasis. Mediators Inflamm. 2017;2017:9624760. 28210073 10.1155/2017/9624760 PMC5292164

[39] Shan X , Zhang C , Wang Z , et al. Prognostic value of a nine‐gene signature in glioma patients based on tumor‐associated macrophages expression profiling. Clin Immunol. 2020;216:108430. 32325251 10.1016/j.clim.2020.108430

[40] Li F , Sun Y , Huang J , Xu W , Liu J , Yuan Z . CD4/CD8 + T cells, DC subsets, Foxp3, and IDO expression are predictive indictors of gastric cancer prognosis. Cancer Med. 2019;8:7330‐7344. 31631566 10.1002/cam4.2596 PMC6885892

[41] Cabrita R , Lauss M , Sanna A , et al. Tertiary lymphoid structures improve immunotherapy and survival in melanoma. Nature. 2020;577:561‐565. 31942071 10.1038/s41586-019-1914-8

[42] Vihervuori H , Autere TA , Repo H , et al. Tumor‐infiltrating lymphocytes and CD8(+) T cells predict survival of triple‐negative breast cancer. J Cancer Res Clin Oncol. 2019;145:3105‐3114. 31562550 10.1007/s00432-019-03036-5 PMC6861359

[43] van der Leun AM , Thommen DS , Schumacher TN . CD8(+) T cell states in human cancer: insights from single‐cell analysis. Nat Rev Cancer. 2020;20:218‐232. 32024970 10.1038/s41568-019-0235-4 PMC7115982

[44] Zhou H , He Y , Li L , Wu C , Hu G . Identification novel prognostic signatures for head and neck squamous cell carcinoma based on ceRNA network construction and immune infiltration analysis. Int J Med Sci. 2021;18:1297‐1311. 33526991 10.7150/ijms.53531 PMC7847625

