# Supplementary figures and images for: Clinical implication and immunological landscape analyses of ANLN in pan‐cancer: A new target for cancer research
Source: Cancer Med. 2022 Aug 28;12(4):4907–20. doi: 10.1002/cam4.5177 (PMC9972146; doi:10.1002/cam4.5177)

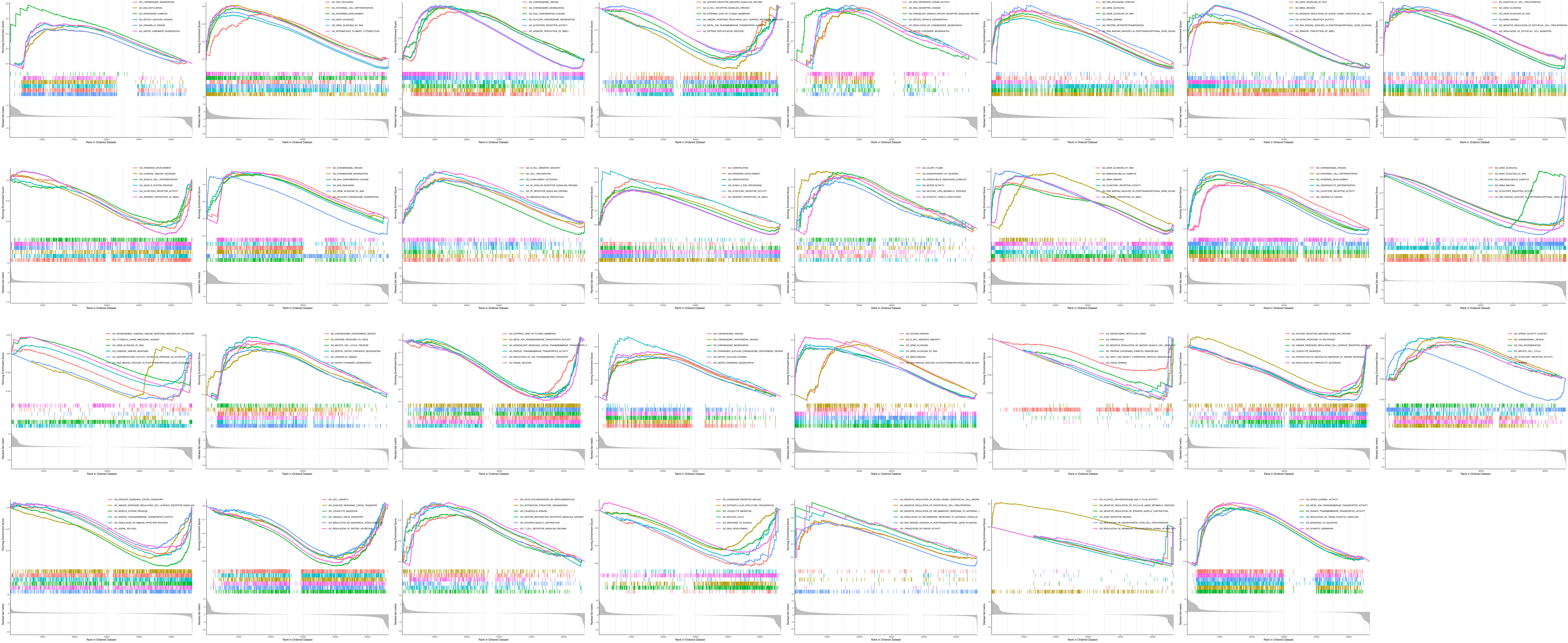

Supplement: Supplementary file 3 — Figure S3 [file CAM4-12-4907-s001.png]
